# Influenza C virus in pre-school children with respiratory infections: retrospective analysis of data from the national influenza surveillance system in Germany, 2012 to 2014

**DOI:** 10.2807/1560-7917.ES.2019.24.10.1800174

**Published:** 2019-03-07

**Authors:** Annemarie Fritsch, Brunhilde Schweiger, Barbara Biere

**Affiliations:** 1 Robert Koch Institute, National Reference Center for Influenza, FG 17 Influenza and Other Respiratory Viruses, Berlin, Germany

**Keywords:** influenza virus, respiratory infections, epidemiology, Germany, Real-Time PCR, hemagglutinin esterase, air-borne infections, laboratory surveillance, molecular methods, sentinel surveillance, viral infections

## Abstract

**Introduction:**

Recent data on influenza C virus indicate a possible higher clinical impact in specified patient populations than previously thought.

**Aim:**

We aimed to investigate influenza C virus circulation in Germany.

**Methods:**

A total of 1,588 samples from 0 to 4 year-old children presenting as outpatients with influenza-like illness (ILI) or acute respiratory infection were analysed retrospectively. The samples represented a subset of all samples from the German national surveillance system for influenza in this age group in 2012–14. The presence of influenza C virus was investigated by real-time PCR. For positive samples, information on symptoms as well as other respiratory virus co-infections was considered. Retrieved influenza C viral sequences were phylogenetically characterised.

**Results:**

Influenza C viral RNA was detected in 20 (1.3% of) samples, including 16 during the 2012/13 season. The majority (18/20) of influenza C-positive patients had ILI according to the European Union definition, one patient had pneumonia. Viruses belonged to the C/Sao Paulo and C/Kanagawa lineages. Most (11/20) samples were co-infected with other respiratory viruses.

**Conclusion:**

Our data are the first on influenza C virus circulation in Germany and notably from a European national surveillance system. The low detection frequency and the identified virus variants confirm earlier observations outside a surveillance system. More virus detections during the 2012/13 season indicate a variable circulation intensity in the different years studied. Influenza C virus can be considered for ILI patients. Future studies addressing its clinical impact, especially in patients with severe disease are needed.

## Introduction

Influenza viruses are a major threat to human health and are therefore in the focus of national and international health authorities. Among these, influenza virus types A and B are the main considered, as they cause annual epidemics with high morbidity and considerable mortality [1]. In contrast, influenza C virus has been regarded as a pathogen of minor relevance, causing mild or clinically unapparent disease [2,3]. Nevertheless, in recent years, detections of influenza C in hospitalised young children with (severe) lower respiratory tract disease were reported [4-9]. Thus, the clinical and epidemiological significance of this virus species might have been underestimated and needs to be reassessed.

In Europe, the burden of influenza C virus infection in children and adults is largely unknown, as no systematic surveillance data are available. The few studies published mainly focus on clinical data, mostly from hospitalised children [4,9,10]. In Germany, no surveillance data and no sequence information on circulating influenza C viruses have ever been reported. Therefore, we decided to search for influenza C in our outpatient sample collection assembled for the purpose of influenza virus surveillance in Germany. As young children are described to have the highest infection rates [6,7,11], we confined our study to the 0–4 year-old age group. We furthermore sequenced the haemagglutinin esterase (HE) gene from influenza C-positive samples to phylogenetically characterise the detected viruses.

## Methods

### Clinical samples

All samples were collected from practitioners participating in the national influenza surveillance, who are distributed over the complete German territory and represent a statistically valid proportion of the German population [12]. These practitioners continuously collect nasal or throat swabs from non-hospitalised patients presenting with symptoms of influenza-like illness (ILI) according to the European Union (EU) definition or an acute respiratory infection (ARI). An ILI case is defined by a sudden disease onset with at least one of four systemic symptoms (fever or feverishness, malaise, headache, myalgia) and at least one of three respiratory symptoms (cough, sore throat, shortness of breath) [13], while ARI is an acute respiratory disease with at least one of the four following symptoms: fever, cough, rhinorrhoea or sore throat. The samples are sent to the German National Influenza Centre, accompanied by a completed questionnaire on patient characteristics, sampling date, disease symptoms, influenza vaccination status and therapeutic intervention i.e. antiviral treatment. They are routinely analysed for influenza virus types A and B, human respiratory syncytial virus (RSV) as well as – since April 2013 – human adenovirus (AdV), metapneumovirus (HMPV) and rhinovirus (HRV). All samples are stored at -80 °C afterwards.

For this study, a subset of 1,588 samples (66.9%) was selected from a total number of 2,377 samples taken from children ≤ 4 years of age in the years 2012–14 as described in the supplementary file (Supplement S1). Briefly, at least every second sample in a chronological order was retrospectively analysed for influenza C virus. To extend the basis for the co-infection data, all influenza C-positive samples were additionally tested for human parainfluenza viruses types 1–4 and coronaviruses OC43, NL63, HKU1 and 229E. Positive samples taken before April 2013 were furthermore retrospectively examined for AdV, HMPV and HRV.

### Ethical statement

The conduct of a sentinel surveillance is covered by German legislation (§13, §14, Protection against Infection Act). The German national surveillance of influenza and other respiratory viruses was furthermore approved by an ethical committee of the Charitè Berlin (application number EA2/126/11). Additionally, for all samples a written consent was given for their inclusion in research studies. All analyses were done with pseudonymised data.

### Sample preparation, nucleic acid extraction and cDNA synthesis

After their arrival in the laboratory, 3mL of cell culture medium (minimum essential medium (MEM) with N-2-hydroxyethylpiperazine-N-2-ethane sulfonic acid (HEPES) buffer with 5,000U/mL PenStrep) was added to the swabs (Copan Diagnostics, Murrieta, United States (US)) to wash out the attached viruses.

RNA was extracted from 200µL sample material using the MagNaPure96 DNA and Viral NA Small Volume Kit (Roche, Basel, Switzerland) and eluted in 50µL elution buffer.

In a total reaction volume of 20µL, 12.5µL of extracted RNA were subjected to cDNA synthesis applying random hexamer primers and 200U Moloney murine leukaemia virus (M-MLV) Reverse Transcriptase (Thermo Fisher Scientific, Waltham, US). Synthesised cDNA was diluted 1:1 with H_2_O to a total volume of 40µL to allow robotic pipetting of 384-well PCR plates.

For sequence analyses, cDNA was synthesised with the AccuScript Hi-Fi Reverse Transcriptase (Agilent, Santa Clara, US) and a primer that binds to the conserved 3’ end of the RNA gene segments (Uni11, see Table 1).

**Table 1:** List of oligonucleotide sequences used in the study

Assay name	Oligonucleotide name	Oligonucleotide sequence (5’–3’)	Amount in nM	Method
FluC NP qPCR	FluC NP F1068	GCRTGCTTTGGRCTTGCTTATG	600	qPCR
	FluC NP R1161	ARTTTCCTATTTTCATTCTGTTTCTCAAC	600	
	FluC NP TM1100	FAM – TTTGGTYTCTGCYATGGTYAGCCAYCCTCT - BHQ1	200	
FCV qPCR (IC)	FCV F54	CGTTACCGCCACACCCAT	300	qPCR
	FCV R141	GAGTTCACGAAAGATTTCAGACCAT	300	
	FCV TM96	LC610 - ACCCATCATTCTAACACTCCCGCCAAT - BHQ1	100	
HE fragment 1	FluC HE F1	AGCAGAAGCAGGGGKTTAATAATG	500	nPCR
	FluC HE F7	AGCAGGGGTTTAATAATGTTTTTCTC	500	
	FluC HE R866	CCAGAATTCCCTGTGTAAGGTGA	500	
	FluC HE R895	ATCATGTCACATTGCATTGTTGG	500	
	FluC HE F477	CCAGAAAARCATYTATGAATTGGC	500	Seq
	FluC HE R353	CCAGGTGGGCCAAACATACT	500	
HE fragment 2	FluC HE F714	GCATCTTGTGGCTTCTTGCTATT	500	nPCR
	FluC HE F722	TGGCTTCTTGCTATTTCATYTATGAYAG	500	
	FluC HE R1474	CTTTTGTYACACCTCCTCCTGAT	500	
	FluC HE R1511	TCATTTCCAATTTTYTCRAAYCC	500	
	FluC HE F1133	ATAAAGAAATGAGGGACYTGCTGT	500	Seq
	FluC HE R1085	ATYARCATGCACCCTGGAGTG	500	
HE fragment 3	FluC HE F1327	ACTGATACCACTGTAACCAAACCYAA	500	nPCR
	FluC HE F1338	TGTAACCAAACCYAARAGCAGRAT	500	
	FluC HE R2067	AGCAAGGGGATTTTTGTTTTTYATAA	500	
	FluC HE R2073	AGCAGTAGCAAGGGGWTTTTTGT	500	
	FluC HE F1703	TGTGGGAACTAGCTTCAGAAATAAC	500	Seq
	FluC HE R1663	AGGCTCTTATTATYCCCAATTCTCC	500	
cDNA synthesis	Uni11	AGCAGAAGCAG	1,000	Seq

BHQ1: black hole quencher 1; FAM: fluorescein amidite; FCV: feline calicivirus; HE: haemagglutinin esterase; IC: internal control; LC610: LightCycler 610; NP: nucleoprotein; nPCR: nested PCR; qPCR: quantitative/real-time PCR; seq: sequencing.

### Real-time PCR 

Real-time PCR (qPCR) for the detection of the influenza C nucleoprotein (NP) gene and feline calicivirus (FCV; internal control) was carried out on LC480II real-time PCR thermal cyclers (Roche, Basel, Switzerland) in a total reaction volume of 15µL in either a 96-well or 384-well plate. The reaction contained 1x PCR buffer, 4mmol/L MgCl_2_, 1mmol/L deoxynucleoside triphosphate (dNTP; Thermo Fisher Scientific, Waltham, US) with deoxyuridine triphosphate (dUTP; GE Healthcare, Chicago, US), 600ng bovine serum albumin (BSA; Thermo Fisher Scientific, Waltham, US), 0.3U (singleplex) or 1U (duplex) Platinum Taq Polymerase (Thermo Fisher Scientific, Waltham, US), primers and probes as listed in Table 1 (Metabion, Planegg, Germany) [14,15], and 5µL of the prediluted cDNA. After 5 min at 95 °C for Taq DNA polymerase activation, a total of 45 cycles consisting of denaturation at 95 °C for 15 s and annealing at 60 °C for 30 s were performed. After the run, data were analysed using the LightCycler software version 1.5.1.62.

### Real-time PCR validation

Assay validation was performed with synthetic double stranded DNA strings (gBlocks; IDT, Skokie, US) in singleplex as well as duplex format (including the internal control FCV) on 96-well as well as 384-well plates.

For the determination of the linear detection range and the correlation (R^2^) of quantification cycle (C_q_) values, a 10-fold dilution series (10^6^–10^1^ copies per reaction) was examined in duplicates. PCR efficiency was calculated by inserting the slope value of the standard curve into the formula E = 10^(-1/slope)-1^.

The limit of detection (LOD) was established as 95% detection probability, calculated by probit analyses of the results of a 10-fold examination of low copy numbers (50–0.1 genome equivalents per reaction) applying the IBM SPSS Statistics 20 software.

For intraassay precision, gBlocks were examined sixfold in a single run, while for interassay precision the intraassay data were extended by two additional runs with double reactions. All reproducibility runs were performed on consecutive days in independent experiments, and precision was described as standard deviation of the observed C_q_ values.

Specificity was ensured by the examination of a number of respiratory viruses (influenza A subtypes A(H1N1)pdm09, A(H1N1) (circulating until 2009), A(H3N2), A(H7N9), A(H5N1), A(H5N8), influenza B, HRV, HMPV, RSV types A and B, adenovirus types B3 and C1, human coronaviruses NL63, 229E, OC43 and MERS coronavirus, parainfluenza virus types 1–4, enterovirus D68) as well as *Staphylococcus aureus* and human genomic DNA.

### Sequence determination

Conventional PCR for sequence determination of the HE gene was carried out in a total reaction volume of 50µL. The reaction contained 1x ExTaq buffer, 1.25mmol/L dNTP (Thermo Fisher Scientific, Waltham, US) with dUTP (GE Healthcare, Chicago, US), 1.25U ExTaq Polymerase (TaKaRa, Kusatsu, Japan), 500nM primers (Metabion, Planegg, Germany) as listed in Table 1, and 5µL of the prediluted cDNA. Alternatively, the SuperScript III One-Step RT-PCR System with Platinum Taq High Fidelity (Thermo Fisher Scientific, Waltham, US) was used without modification of reaction conditions.

For each sample, three overlapping nested PCRs yielded amplicons that span the HE gene. They were sequenced after purification (MSB Spin PCRapace Kit and Invisorb Spin DNA Extraction Kit, Stratec biomedical, Birkenfeld, Germany) using the dye terminator chemistry (ABI-Prism Big Dye Terminators v3.1 Cycle Sequencing Kit, Thermo Fisher Scientific, Waltham, US) in a 3130xl Genetic Analyzer (Thermo Fisher Scientific, Waltham, US). Additional ‘internal’ sequencing primers (labelled ’Seq’ in Table 1) were used for amplicon sequencing in cases where the nested PCR primers did not yield a sequence spanning the complete amplicon. All HE sequences were processed and assembled in the Geneious software before their deposition at the Global Initiative on Sharing All Influenza Data (GISAID; www.gisaid.org) database (EPI1183982–EPI1183998). The applied amino-acid numbering includes the signal peptide.

### Phylogenetic analyses

All HE sequence analyses were performed with Geneious version 10.0.5. Multiple sequence alignments were compiled on the basis of the MAFFT algorithm. The N-terminal sequences including the signal peptide sequence (MFFSLLLMLGLTEA [16]) as well as the C-terminal region with incomplete sequence information (last 13 nt including the stop codon) were excluded. The alignment for phylogenetic analyses thus covered the nt 43 to 1,955 of the complete coding sequence and was calculated including reference sequences downloaded from the GISAID database (see Supplement S2). Maximum likelihood trees were constructed applying the HKY85 model and the SPR tree topology search. Branching reliability was estimated by performing 1,000 bootstrap replicates. Trees were manually edited in Corel Draw X6.

## Results

A qPCR assay for the detection of influenza C viruses was established as singleplex as well as duplex qPCR including our routine internal control FCV. The assay proved to be a robust and sensitive tool and furthermore did not show any cross-reactivity to a variety of viral respiratory pathogens and to human genomic DNA (validation results summarised in Table 2).

**Table 2:** Validation results for the influenza C qPCR assay

Assay	Slope	E	R^2^	LOD (geq)	Reproducibility					
					Intraassay			Interassay		
					500,000	5,000	50	500,000	5,000	50
FluC	-3.58	90%	0.998	10.5	0.09	0.04	0.50	0.19	0.28	0.47
FluC + FCV (96-well)	-3.32	100%	0.997	10.3	0.04	0.02	0.39	0.44	0.49	0.60
FluC + FCV (384-well)	-3.35	99%	0.9895	13.5	0.14	0.03	0.32	0.22	0.17	0.32

qPCR: quantitative/real-time PCR; E: PCR efficiency; geq: genome equivalents; R^2^: correlation coefficient; LOD: limit of detection.

**Figure 1:**
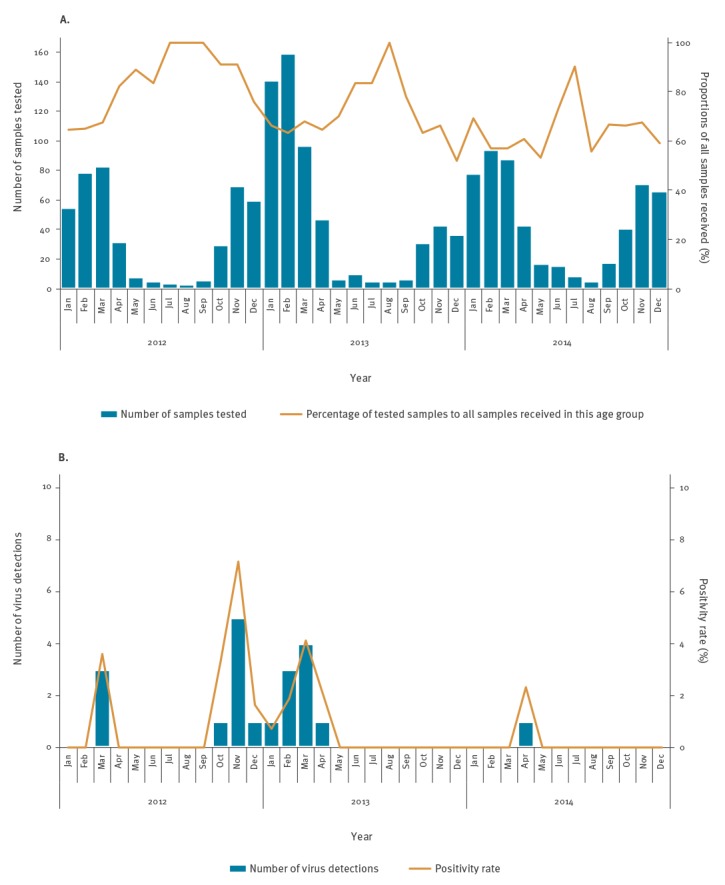
Monthly distribution in 0–4 year-old children of (A) the number of samples tested for influenza C, as well as testing coverage among samples received by the surveillance system (B) the number of positive samples for influenza C and resulting positivity rate, Germany, 2012–2014 (n =1,570)^a^

**Figure 2:**
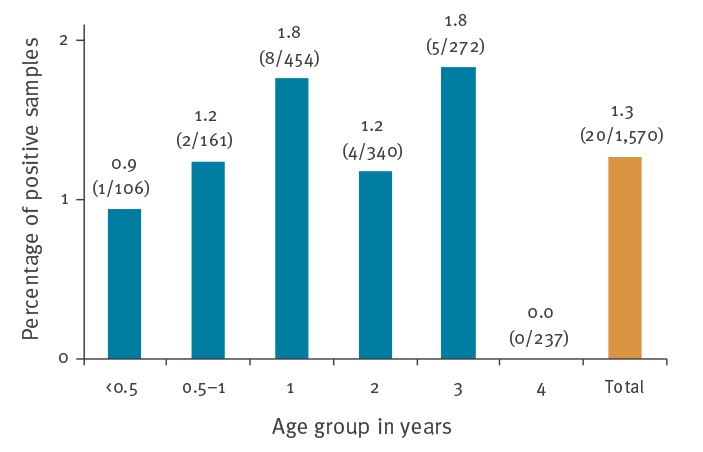
Influenza C positivity rates in samples in 0–4 year-old children by age group, Germany, 2012–2014 (n = 1,570 samples)^a^

The duplex qPCR approach was applied to retrospectively examine 1,588 throat or nasal swabs, of which 1,570 samples gave valid qPCR results, i.e. yielded either an influenza C or a FCV signal (or both) in duplex qPCR runs. Twenty samples (1.3%) were found positive for influenza C virus RNA, with C_q_ values ranging from 19 to 39. The positive samples predominantly were taken between October 2012 and April 2013, reaching an average positivity rate of 2.6% (16/604) in these months (0.7–7.1% per month, Figure 1). Outside of this particular winter season, viruses were identified only sporadically with detection rates of 1.2% (3/249, January–April 2012) or 0.2% (1/414, October 2013–April 2014). No virus detection was achieved from May to September of any year studied. Also, no particular age distribution could be observed (Figure 2).

Samples are ascribed to a month according to their sampling date.

^a^ A total of 1,588 throat or nasal swabs were considered. Of these, the 1,570 samples that gave a valid quantitative/real-time PCR result are shown in the Figure.

Percentages as well as total numbers (positive/tested) are depicted above the chart bars.

^a^ A total of 1,588 throat or nasal swabs were considered. Of these, the 1,570 samples that gave a valid quantitative/real-time PCR result are shown in the Figure.

All influenza C-positive samples were additionally examined by qPCR to identify other respiratory viruses. More than half of the influenza C-positive patients (11/20; 55%) proved to be co-infected with diverse other respiratory pathogens (Table 3), with influenza C C_q_ values covering the complete range of 19 to 39.

**Table 3:** Summary of patient and virus characteristics for influenza C-positive samples, Germany, 2012–2014 (n = 20 samples)

Sample ID	Sex	Age	Collection date month-year	Collected in	Symptoms	FluC C_q_	Lineage	Isolate ID^a^	Co-infection (C_q_)
12–02332^b^	M	2y	Mar-12	Thuringia	ILI	27	SP	EPI_ISL_300530	None
12–02562	M	3y	Mar-12	Thuringia	ILI	21	SP	EPI_ISL_300531	PIV-3 (28)
12–02741	M	3y	Mar-12	Saxony	ILI	19	Ka	EPI_ISL_300532	Influenza A(H3N2) (24)
13–00167	M	20m	Oct-12	Thuringia	ILI	19	SP	EPI_ISL_300533	None
13–00231^b^	F	18m	Nov-12	Bremen	ILI	35	NA	NA	PIV-3 (26), AdV (34)
13–00344^b^	F	3m	Nov-12	North Rhine-Westphalia	ILI	22	SP	EPI_ISL_300534	HRV (24)
13–00418	M	2y	Nov-12	Hamburg	ILI	25	SP	EPI_ISL_300535	None
13–00498	M	3y	Nov-12	Schleswig-Holstein	ILI	30	SP	EPI_ISL_300536	CoV OC43 (25)
13–00580^b^	M	13m	Nov-12	Thuringia	ILI	19	SP	EPI_ISL_300537	None
13–00631	F	3y	Dec-12	Baden-Wuerttemberg	ILI	35	SP	EPI_ISL_300538	AdV (22), PIV-3 (30)
13–01570^b^	M	11m	Jan-13	North Rhine-Westphalia	ILI	36	NA	NA	None
13–03024	M	23m	Feb-13	Berlin	ARI	29	SP	EPI_ISL_300539	None
13–03232	F	8m	Feb-13	Rhineland-Palatinate	ILI	39	NA	NA	RSV-A (23)
13–04022	M	19m	Feb-13	North Rhine-Westphalia	ILI	29	SP	EPI_ISL_300540	None
13–04332	M	2y	Mar-13	Brandenburg	ILI	25	SP	EPI_ISL_300541	Influenza B(Yam) (30)
13–04588	F	2y	Mar-13	Hesse	ILI, pneu	21	SP	EPI_ISL_300542	Influenza A(H3N2) (35)
13–04691^b^	F	18m	Mar-13	Brandenburg	ILI	25	SP	EPI_ISL_300543	HRV (32)
13–05206	M	13m	Mar-13	Brandenburg	ILI	26	SP	EPI_ISL_300544	None
13–05486	F	12m	Apr-13	Brandenburg	ARI	33	SP	EPI_ISL_300545	None
14–03242	M	3y	Apr-14	Rhineland-Palatinate	ILI	32	Ka	EPI_ISL_300546	AdV (19)

AdV: adenovirus; ARI: acute respiratory infection; CoV: coronavirus; C_q_: quantification cycle; F: female; FluC: influenza C virus real-time PCR; HRV: human rhinovirus; GISAID: Global Initiative on Sharing All Influenza Data; ILI: influenza-like illness; Ka: Kanagawa; M: male; m: months; NA: not available (the sequence could not be obtained); PIV: parainfluenza virus; pneu: pneumonia; RSV: respiratory syncytial virus; SP: Sao Paulo; Yam: Yamagata lineage; y: years.

^a^ Deposited at Global Initiative on Sharing All Influenza Data (GISAID).

^b^ ‘Group(add)’ sample according to sampling scheme explained in Supplement S1.

All 20 patients with influenza C virus infection reported fever and cough. Fifteen patients reported a maximum temperature between 38.5 °C and 40.2 °C, while for the remaining five patients the maximum temperature was not provided. Additionally, a sudden disease onset (18/20), rhinitis (18/20), sore throat (8/20) and muscular pain and/or headache (6/20) were predominant symptoms. Clinical signs of pneumonia were reported for one patient with an influenza C C_q_ value of 25, but also low amounts of influenza A(H3N2) were detected in this sample. In patients with a sole influenza C virus infection, the sudden disease onset (7/9), the maximum fever (38.9 °C – 40.2 °C in 6 patients), rhinitis (8/9), sore throat (4/9) and muscular pain and/or headache (2/9) were reported in similar proportions.

The sequencing of the HE gene was achieved for 17 samples, of which three yielded only partial sequences. Two of the incomplete sequences covered a consecutive stretch of 1,071 and 1,218 nt, respectively, while the third sequence gave two individual fragments at the N- and C-terminus of the viral protein. With a length of 1,899 – 2,005 nt, all other sequences reached nearly the full length of the HE protein, and were therefore included into further analyses.

**Figure 3:**
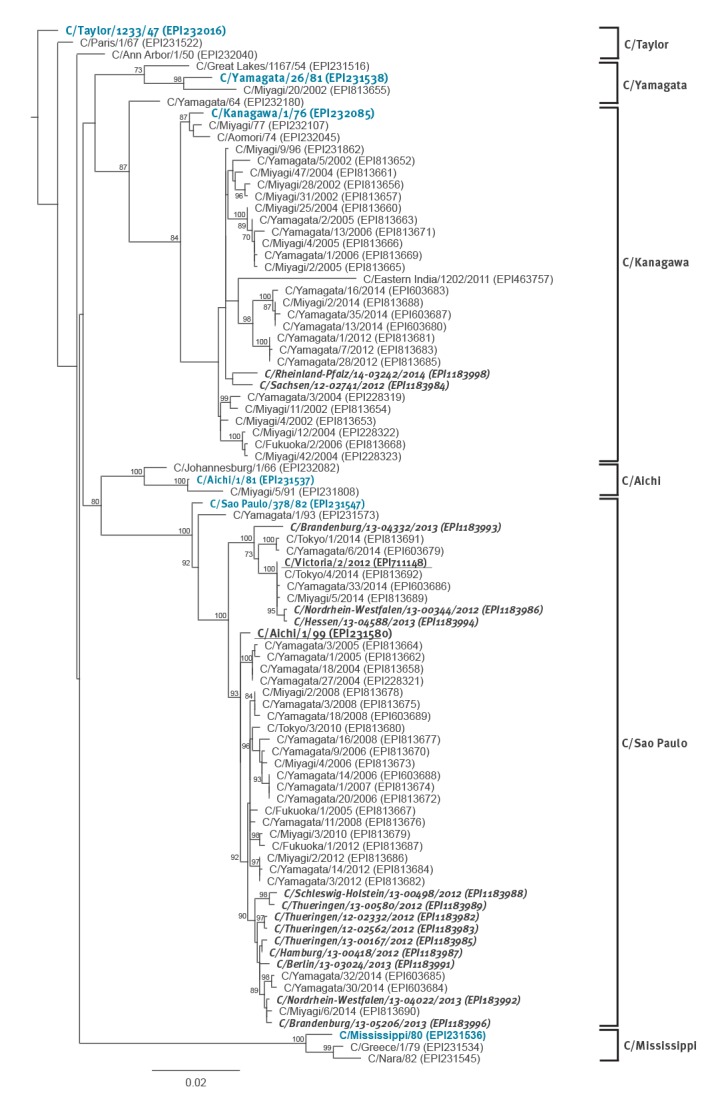
Phylogenetic analyses of haemagglutinin esterase genetic sequences derived from influenza C-positive samples in 0–4 year-old children using a set of representative sequences from different lineages, Germany, 2012–2014

Phylogenetically, the vast majority of samples (12/14) clustered into both subclades of the C/Sao Paulo lineage represented by C/Aichi/1/99 and C/Victoria/2/2012 (Figure 3). Nine sequences add to a C/Aichi/1/99 subgroup of only three samples from Japan, represented by C/Miyagi/6/2014 and – within the Sao Paulo lineage – characterised by the amino-acid substitution K204N. These sequences were derived from samples collected between March 2012 and March 2013. Three sequences, dating from between November 2012 and March 2013, group into the C/Victoria/2/2012 clade that, within the Sao Paulo lineage, are sole carriers of the amino-acid substitution Q372K. However, both amino-acid substitutions K204N and Q372K can also be found in other influenza C lineages. Two of our viral sequences sampled in March 2012 and April 2014 cluster into the Kanagawa-lineage and form a separate small subgroup carrying the amino acid substitution K75R.

Bootstrap analyses were performed applying the maximum likelihood algorithm with 1,000 replicates as described in the methods section. The tree is rooted at C/Taylor/1233/47. Only bootstrap values greater than 70 are displayed at the branch nodes. Prototypes of influenza C virus lineages are in bold font and blue colour, subclade representatives are bold and underlined. The German sequences are marked in bold italic letters. The scale indicates the average substitutions per site. Accession numbers of all sequences are provided in brackets.

The three incomplete sequences were only characterised based on the nt homologies to other sequences. The two fragments of sample 13–04691 (775nt, 557nt) are 100% identical to our sample sequence 13–04332, which belongs to the C/Victoria/2/2012 subgroup of the C/Sao Paulo clade. Similarly, sample sequence 13–05486 (1,037nt) is 100% identical to the sequence of C/Sao Paulo samples 13–04022 and 13–05206, which group into the C/Miyagi/6/2014 subgroup of the C/Aichi/1/99 subclade. Sample sequence 13–00631 has a similarity of > 99.2% to the same Sao Paulo lineage cluster, while the similarity to C/Victoria/2/2012 (98.1%) and the prototype strain sequences for the other five HE lineages is lower (≤ 95.4%).

## Discussion

Although influenza C virus was discovered 70 years ago, there is only little knowledge on the biology and epidemiology of this virus type. Some studies indicated a low clinical impact with only mild symptoms [2,3,17], and in spite of a high seroprevalence in the population, virus detections were rare [6,11,18]. These findings led to the conclusion that influenza C infection is common, but clinically inapparent or too mild to require a visit to a doctor [2]. Additionally, the low detection rate may be in part due to the fact that in earlier times virus diagnostics were mainly based on virus culture, which is difficult for influenza C [6,19] and necessitates conditions that differ from influenza A and B virus cultivation [20]. As a consequence, influenza C virus diagnostics were restricted to specialised laboratories and correspondingly rare [4].

With the introduction of molecular methods, influenza C has been increasingly included into studies on respiratory pathogens and clinical diagnostics. Thereby, the low detection rates in the general population were confirmed, but a higher clinical impact for paediatric patients was indicated, as influenza C was described to also cause lower respiratory tract disease [6-10]. In a 6-month prospective study (December 2009–May 2010) including Japanese children with community-acquired pneumonia, bronchiolitis or bronchitis, influenza C infection was identified even with a prevalence approximating those of influenza A or HMPV [5]. Further studies, mostly in children, described the symptoms of influenza C infection to be indistinguishable from influenza A and B infections [4,6], although the maximum body temperature may be lower and the fever shorter compared with influenza A [6,21,22]. In Finnish military recruits, influenza C virus caused common cold-like symptoms, but occasionally resulted in pneumonia and bronchitis [3].

For Europe, only little information on influenza C circulation has been published. In adults, a seroprevalence of ca 70% and more was found in France [23], Finland [3], and United Kingdom [17]. Applying PCR on samples from all age groups, a virus detection rate of ≤ 1% was reported for Normandy/France [10], Scotland [24] and Spain [21], but higher detection rates of 3.5 – 4.2% were found in two adult studies from Finland [3,25]. Outside Europe, a similar seroprevalence as well as comparable detection rates have been described for Australia, Canada, Japan, Nigeria, the Philippines, Peru and the US [6-8,11,15,18,19,22,26-28].

In view of the lack of knowledge on influenza C virus circulation in Germany, we decided to generate the first systematic data on the basis of our national influenza virus surveillance. We chose to examine the age group of 0–4 years, as young children have been shown to have the highest infection rates [6,7,11,19]. We analysed a representative subset of the 2,377 samples received in this age group between 2012 and 2014 (52.1 – 100% of all samples in the corresponding month).

First, we validated a previously published qPCR [15] and duplexed it with our routine internal control, FCV. In an extensive validation effort according to international standards [29], we found the singleplex as well as the duplex format to perform with high sensitivity, specificity and precision. We therefore applied it to our sample compilation and identified influenza C RNA in 20 of 1,570 samples with valid qPCR results (1.3%). The vast majority of virus detections (16 of 20) was found in samples that were collected between October 2012 and April 2013, signalling a more pronounced virus circulation during these months with positivity rates of up to 7.1% (5/70) in November 2012. As these samples were collected in 10 of 16 German federal states, virus circulation was not confined to a region, but widespread, maybe even nationwide. The virus prevalence however was markedly lower during the other winter seasons observed in this study, and no virus could be detected during the summer months. This absence of summer circulation is in congruence with reports from Japan, France, Finland and Spain [3,4,6,10,25], but is in contradiction to a report from Catalonia in Spain, in which the majority of positive samples were taken in August and September of the observed time span [21]. An upsurge of influenza C virus circulation in the spring of 2013 was also observed in the Philippines [8], but did not occur in Japan, from where virus circulation in even numbered years was reported, including the years 2012 and 2014 [11,30]. However, a biennial pattern of virus circulation with increased or time-shifted profile has also been described for other respiratory viruses in Germany [31,32] and therefore is conceivable, but remains open in our study due to the short study period, which presents a limitation. In total, the proportion of influenza C-positive patients was small, but within the expected range. It needs to be emphasised though, that the obtained overall positivity rate is largely based on only few months during the winter season 2012/13 with substantial virus circulation in our study population.

Because of our limited access to clinical data, only few conclusions can be drawn with regard to the clinical relevance of influenza C virus. The vast majority of patients (18/20) carrying the virus fulfilled the EU ILI definition. Although, due to our study design, there may be a bias to ILI cases during periods of influenza A and B virus circulation, our findings are in concordance with other studies, in which ILI was described for the majority or all of influenza C infected patients [21,27]. Bronchitis or bronchiolitis was not reported for any patient, but one child (infected also with an influenza A virus) presented with symptoms of pneumonia. However, the proportion of pneumonia in our influenza C-positive samples does not differ considerably from that of our complete sample collection of this age group spanning the years 1999 to 2017 (data not shown). In our ambulant setting, we thus do not see an indication for an accumulation of lower respiratory tract disease in influenza C infected patients, but an influenza-like clinical presentation is common.

Interestingly, a substantial share of influenza C-positive samples showed co-infection with other pathogens, as reported also in other studies [3,4,15,27]. In these cases, the cause for ILI symptoms cannot clearly be attributed to influenza C. Yet, we used qPCR assays with comparable performance characteristics (LOD and efficiency), so that a comparison of the obtained C_q_ values can be semiquantitatively interpreted for the different pathogens within one sample. In our study, the majority of co-infected samples (6/11; 54.5%) exhibited the highest viral load for influenza C, including the pneumonia case for whom it was ca 10,000-fold higher than that of influenza A at the time point and the sample site examined. In four samples, the influenza C C_q_ was close to the detection limit (≥ 35) and thus influenza C was presumably of minor relevance. Repetitive sampling from the same patients and continuous parallel assessment of the patients clinical presentation could clarify the role of the single pathogens in the disease course, but is not included in our routine influenza surveillance system. Therefore, we cannot judge on the temporal dynamics of virus replication and the clinical impact of each virus detected.

Due to the slow evolution and thus high antigenic homology of influenza C virus [11], we decided to characterise the German sequences only on the basis of the HE gene sequences. The HE glycoprotein has a variety of functions in the viral replication cycle and greatly determines the antigenicity of the virus [33]. Based on antigenic and phylogenetic characteristics of this protein, distinct virus lineages have been described that were named after their prototype strains C/Taylor/1233/47, C/Kanagawa/1/76, C/Mississippi/80, C/Aichi/1/81, C/Yamagata/26/81 and C/Sao Paulo/378/82 [30,34]. All influenza C lineage clusters are comprised of isolates from a multitude of continents, indicating a global circulation of virus lineages [34]. However, four lineages seemingly disappeared (C/Taylor, C/Aichi, C/Mississippi, C/Yamagata), and only the C/Kanagawa and C/Sao Paulo lineage have been detected within the last decade [8,9,19,21,27,30,34].

From our positive samples, a total of 17 partial and near full-length HE sequences could be generated. We almost exclusively detected C/Sao Paulo lineage viruses, and only two C/Kanagawa viruses were identified. Our C/Sao Paulo sequences add to both lineage subclades described by Matsuzaki et al. and represented by C/Aichi/1/99 and C/Victoria/2/2012 [30]. A total of 11 sequences (9 complete, 2 incomplete) group into the C/Aichi/1/99 subclade and were sampled between March 2012 and April 2013, while three additional sequences (2 complete, 1 incomplete) group into the C/Victoria/2/2012 subclade and were sampled between November 2012 and March 2013. Thus, viruses of both subclades co-circulated during the 2012/13 winter season. In contrast, the two C/Kanagawa lineage viruses were sampled in March 2012 and April 2014, thus outside of the period with increased infection rates. They form a distinct cluster within the C/Kanagawa clade, most closely related to C/Miyagi/9/96. Both C/Kanagawa viruses are almost identical to each other showing a nt homology of 99.1%, although they were sampled with a 2-year distance. Their closest neighbour, C/Miyagi/9/96 even has a homology of 99.5% and 99.3% on the nt level. This further supports the described genetic stability of this virus type compared with influenza A and B viruses [30], possibly also reflecting their antigenic properties.

To summarise, our study is the first report on influenza C circulation in the context of a nationwide outpatient influenza surveillance system in Europe. We found influenza C in a proportion of samples that was in accordance with previous reports. An increased and widespread virus circulation was observed during the winter and spring months of 2012/13, with viruses predominantly belonging to C/Aichi/1/99 subclade of C/Sao Paulo lineage viruses. Infected patients showed symptoms of ILI including upper as well as lower respiratory tract infection, although its association to the observed clinical symptoms remain uncertain in the majority of cases due to the identified co-infections. Further knowledge is needed about the virus epidemiology, its transmission patterns, its role in sole and mixed infections as well as the associated disease burden, especially in young children and patients with lower respiratory tract disease.

## Supplementary Data

307000 Supplement S1

## Supplementary Data

92000 Supplement S2
