# Supplementary material for: Influenza C virus in pre-school children with respiratory infections: retrospective analysis of data from the national influenza surveillance system in Germany, 2012 to 2014
Source: Euro Surveill. 2019 Mar 7;24(10):1800174. doi: 10.2807/1560-7917.ES.2019.24.10.1800174 (PMC6415498; doi:10.2807/1560-7917.ES.2019.24.10.1800174)
Supplement: Supplement S1 [file 1800174_BIERE_SupplementS1.pdf]

Supplement 1

Fritsch et al.
I Influenza C virus in pre-school children with respiratory infections: retrospective analysis of data from the national influenza surveillance system in Germany, 2012 to 2014

This supplementary material is hosted by Eurosurveillance as supporting information alongside the article "Influenza C virus in pre-school children with respiratory infections: retrospective analysis of data from the national influenza surveillance system in Germany, 2012 to 2014" on behalf of the authors who remain responsible for the accuracy and appropriateness of the content. The same standards for ethics, copyright, attributions and permissions as for the article apply. Eurosurveillance is not responsible for the maintenance of any links or email addresses provided therein

Sample selection

In our study period, we received a total of 2,377 samples that fulfilled our study criteria. Every second sample from a chronologically sorted database list (date of sample arrival in the laboratory) was included in our study, regardless of earlier examination results. These 1,188 samples are therefore considered to be representative for the complete sample collection (group(rep)).

In view of the presumably low detection rates, and assuming that the detection probability might be higher in negatively pre-tested samples, we additionally included 438 non-group(rep) samples (group(add)) that had all been pretested negative for other respiratory viruses in our routine procedures (influenza A and B, RSV in the complete study period; AdV, HRV and HMPV starting April 2013). Thereby we aimed to identify additional influenza C positive samples for HE gene sequencing, in order to get further information on virus diversity.

Of the 1,626 samples picked from our database, material was available for 1,588 samples (1,162 group(rep) samples, 426 group(add) samples).

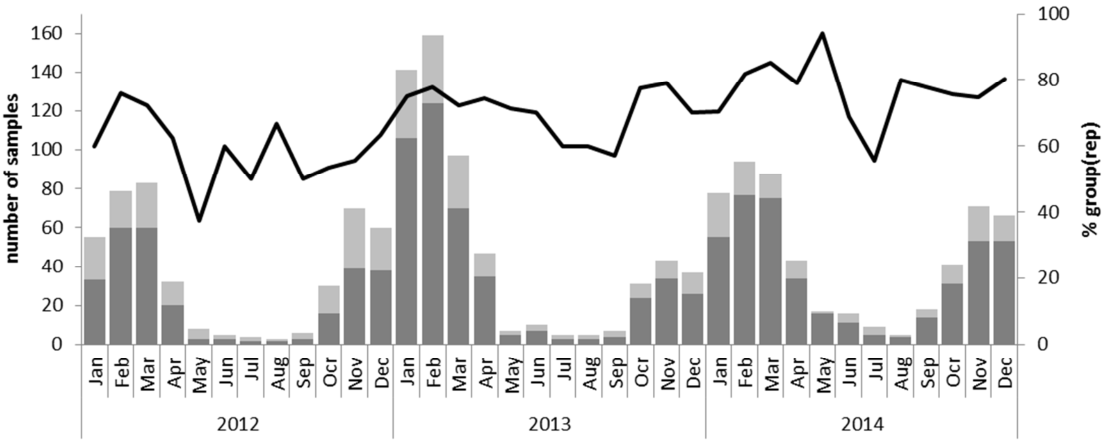

Supplementary Figure S1
Sample numbers are depicted as dark grey (group(rep)) or light grey (group(add)) bar. On the secondary axis, the percentage of group(rep) samples corresponding to the total sample number examined is shown as black line. Samples are ascribed to a month according to their sampling date.

Results

In both sample subsets influenza C virus RNA could be detected: 14 group(rep) and 6 group(add) samples proved to be positive, thus yielding average positivity rates of 1.22% and 1.42%, respectively. Positivity rates varied between 0% and 6.3% for group(rep) and between 0% and 9.7% for group(add). The overall pattern of virus circulation was very similar for both subsets, being the highest during the winter season 2012/13.

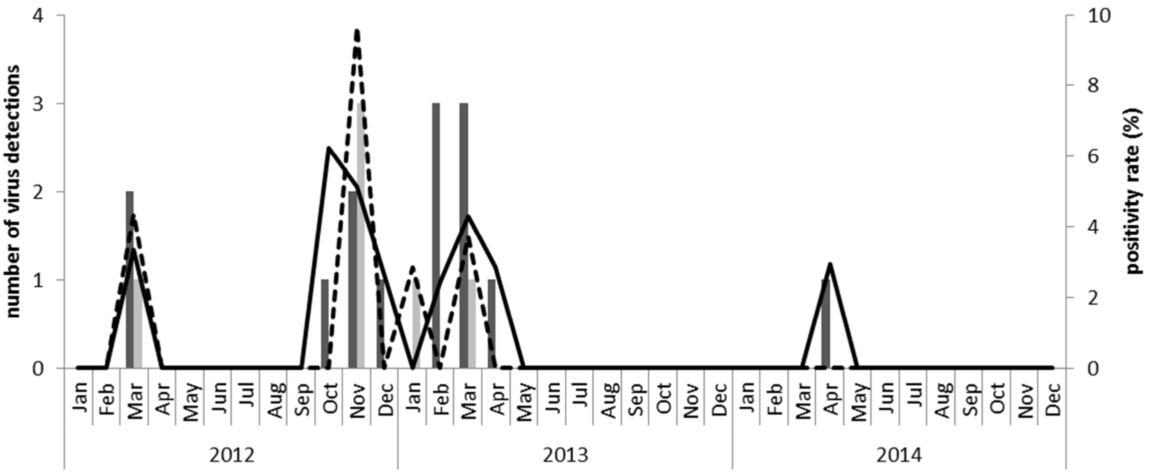

Supplementary Figure S2
Virus detections are shown as dark grey (group(rep)) or light grey (group(add)) bar. On the secondary axis, the positivity rates of group(rep) (continuous line) and group(add) (dotted line) samples are depicted. Samples are ascribed to a month according to their sampling date.

The HE gene sequencing was achieved for 13 group(rep) and 4 group(add) samples. Both C/Kanagawa lineage viruses were detected in the group(rep) samples.

In total, the results for both subsets are very similar. In both groups the detection frequency for influenza C virus was on the expected low level, hindering a conclusive interpretation of potential differences in virus occurrence in the defined subsets. Based on the argument that, in total, we do have a representative sample collection with 73% group(rep) samples with valid qPCR results, the overall analyses were conducted for the complete sample set without any differentiation of the results for group(rep) and group(add) samples.

In the main document, the influenza C positive group(add) samples are labelled in Table 3.
