# Supplementary material for: Influenza C virus in pre-school children with respiratory infections: retrospective analysis of data from the national influenza surveillance system in Germany, 2012 to 2014
Source: Euro Surveill. 2019 Mar 7;24(10):1800174. doi: 10.2807/1560-7917.ES.2019.24.10.1800174 (PMC6415498; doi:10.2807/1560-7917.ES.2019.24.10.1800174)
Supplement: Supplement S2 [file 1800174_BIERE_SupplementS2.pdf]

This supplementary material is hosted by Eurosurveillance as supporting information alongside the article "Influenza C virus in pre-school children with respiratory infections: retrospective analysis of data from the national influenza surveillance system in Germany, 2012 to 2014" on behalf of the authors who remain responsible for the accuracy and appropriateness of the content. The same standards for ethics, copyright, attributions and permissions as for the article apply. Eurosurveillance is not responsible for the maintenance of any links or email addresses provided therein

We acknowledge the authors, originating and submitting laboratories of the sequences from GISAID's EpiFlu™ Database on which this research is based. The list is detailed below.

All submitters of data may be contacted directly via the GISAID website ***www.gisaid.org***

Segment ID	Segment	Country	Collection date	Isolate name	Originating Lab	Submitting Lab	Authors
EPI228319	HE	Japan	2004-Apr-16	C/Yamagata/3/2004		Other Database Import	Matsuzaki,Y.; Sugawara,K.; Furuse,Y.; Shimotai,Y.; Hongo,S.; Oshitani,H.; Mizuta,K.; Nishimura,H.
EPI228321	HE	Japan	2004-May-24	C/Yamagata/27/2004		Other Database Import	Matsuzaki,Y.; Sugawara,K.; Furuse,Y.; Shimotai,Y.; Hongo,S.; Oshitani,H.; Mizuta,K.; Nishimura,H.
EPI228322	HE	Japan	2004-Feb-27	C/Miyagi/12/2004		Other Database Import	Matsuzaki,Y.; Sugawara,K.; Furuse,Y.; Shimotai,Y.; Hongo,S.; Oshitani,H.; Mizuta,K.; Nishimura,H.
EPI228323	HE	Japan	2004-May-27	C/Miyagi/42/2004		Other Database Import	Matsuzaki,Y.; Sugawara,K.; Furuse,Y.; Shimotai,Y.; Hongo,S.; Oshitani,H.; Mizuta,K.; Nishimura,H.
EPI231516	HE	United States	1954-Jan-01	C/Great Lakes/1167/54		Other Database Import	Buonagurio,D.A.; Nakada,S.; Desselberger,U.; Krystal,M.; Palese,P.
EPI231522	HE	France	1967-Jan-01	C/Paris/1/67		Other Database Import	Matsuzaki,Y.; Sugawara,K.; Furuse,Y.; Shimotai,Y.; Hongo,S.; Oshitani,H.; Mizuta,K.; Nishimura,H.
EPI231534	HE	Greece	1979-Jan-01	C/Greece/1/79		Other Database Import	Matsuzaki,Y.; Sugawara,K.; Furuse,Y.; Shimotai,Y.; Hongo,S.; Oshitani,H.; Mizuta,K.; Nishimura,H.
EPI231536	HE	United States	1980-Jan-01	C/Mississippi/80		Other Database Import	Buonagurio,D.A.; Nakada,S.; Desselberger,U.; Krystal,M.; Palese,P.
EPI231537	HE	Japan	1981-Jan-01	C/Aichi/1/81		Other Database Import	Matsuzaki,Y.; Sugawara,K.; Furuse,Y.; Shimotai,Y.; Hongo,S.; Oshitani,H.; Mizuta,K.; Nishimura,H.
EPI231538	HE	Japan	1981-Mar-18	C/Yamagata/26/81		Other Database Import	Matsuzaki,Y.; Sugawara,K.; Furuse,Y.; Shimotai,Y.; Hongo,S.; Oshitani,H.; Mizuta,K.; Nishimura,H.

Segment ID	Segment	Country	Collection date	Isolate name	Originating Lab	Submitting Lab	Authors
EPI231545	HE	Japan	1982-Jan-01	C/Nara/82		Other Database Import	Adachi,K.; Kitame,F.; Sugawara,K.; Nishimura,H.; Nakamura,K.
EPI231547	HE	Brazil	1982-Jan-01	C/Sao Paulo/378/82		Other Database Import	Matsuzaki,Y.; Sugawara,K.; Furuse,Y.; Shimotai,Y.; Hongo,S.; Oshitani,H.; Mizuta,K.; Nishimura,H.
EPI231573	HE	Japan	1993-Apr-30	C/Yamagata/1/93		Other Database Import	Matsuzaki,Y.; Sugawara,K.; Furuse,Y.; Shimotai,Y.; Hongo,S.; Oshitani,H.; Mizuta,K.; Nishimura,H.
EPI231580	HE	Japan	1999-Apr-07	C/Aichi/1/99		Other Database Import	Matsuzaki,Y.; Sugawara,K.; Furuse,Y.; Shimotai,Y.; Hongo,S.; Oshitani,H.; Mizuta,K.; Nishimura,H.
EPI231808	HE	Japan	1991-Feb-25	C/Miyagi/5/91		Other Database Import	Matsuzaki,Y.; Sugawara,K.; Furuse,Y.; Shimotai,Y.; Hongo,S.; Oshitani,H.; Mizuta,K.; Nishimura,H.
EPI231862	HE	Japan	1996-Jun-03	C/Miyagi/9/96		Other Database Import	Matsuzaki,Y.; Sugawara,K.; Furuse,Y.; Shimotai,Y.; Hongo,S.; Oshitani,H.; Mizuta,K.; Nishimura,H.
EPI232016	HE		1947-Jan-01	C/Taylor/1233/47		Other Database Import	Buonagurio,D.A.; Nakada,S.; Fitch,W.M.; Palese,P.
EPI232040	HE	United States	1950-Jan-01	C/Ann Arbor/1/50		Other Database Import	Muraki,Y.; Washioka,H.; Sugawara,K.; Matsuzaki,Y.; Takashita,E.; Hongo,S.
EPI232045	HE	Japan	1974-Jan-01	C/Aomori/74		Other Database Import	Matsuzaki,Y.; Sugawara,K.; Furuse,Y.; Shimotai,Y.; Hongo,S.; Oshitani,H.; Mizuta,K.; Nishimura,H.
EPI232082	HE	South Africa	1966-Jan-01	C/Johannesburg/1/66		Other Database Import	Zimmer,G.
EPI232085	HE	Japan	1976-Jan-01	C/Kanagawa/1/76		Other Database Import	Matsuzaki,Y.; Sugawara,K.; Furuse,Y.; Shimotai,Y.; Hongo,S.; Oshitani,H.; Mizuta,K.; Nishimura,H.
EPI232107	HE	Japan	1977-Jan-01	C/Miyagi/77		Other Database Import	Matsuzaki,Y.; Sugawara,K.; Furuse,Y.; Shimotai,Y.; Hongo,S.; Oshitani,H.; Mizuta,K.; Nishimura,H.
EPI232180	HE	Japan	1964-Jan-01	C/Yamagata/64		Other Database Import	Matsuzaki,Y.; Sugawara,K.; Furuse,Y.; Shimotai,Y.; Hongo,S.; Oshitani,H.; Mizuta,K.; Nishimura,H.

Segment ID	Segment	Country	Collection date	Isolate name	Originating Lab	Submitting Lab	Authors
EPI463757	HE	India	2011-Jul-15	C/Eastern India/1202/2011		Other Database Import	Roy Mukherjee,T.; Chawla-Sarkar,M.
EPI603679	HE	Japan	2014-Apr-16	C/Yamagata/6/2014		Other Database Import	Tanaka,S.; Aoki,Y.; Matoba,Y.; Yahagi,K.; Mizuta,K.; Itagaki,T.; Katsushima,F.; Katsushima,Y.; Matsuzaki,Y.; Sugawara,K.; Furuse,Y.; Shimotai,Y.; Hongo,S.; Oshitani,H.; Mizuta,K.; Nishimura,H.
EPI603680	HE	Japan	2014-May-14	C/Yamagata/13/2014		Other Database Import	Tanaka,S.; Aoki,Y.; Matoba,Y.; Yahagi,K.; Mizuta,K.; Itagaki,T.; Katsushima,F.; Katsushima,Y.; Matsuzaki,Y.; Sugawara,K.; Furuse,Y.; Shimotai,Y.; Hongo,S.; Oshitani,H.; Mizuta,K.; Nishimura,H.
EPI603683	HE	Japan	2014-May-21	C/Yamagata/16/2014		Other Database Import	Tanaka,S.; Aoki,Y.; Matoba,Y.; Yahagi,K.; Mizuta,K.; Itagaki,T.; Katsushima,F.; Katsushima,Y.; Matsuzaki,Y.; Sugawara,K.; Furuse,Y.; Shimotai,Y.; Hongo,S.; Oshitani,H.; Mizuta,K.; Nishimura,H.
EPI603684	HE	Japan	2014-Jun-04	C/Yamagata/30/2014		Other Database Import	Tanaka,S.; Aoki,Y.; Matoba,Y.; Yahagi,K.; Mizuta,K.; Itagaki,T.; Katsushima,F.; Katsushima,Y.; Matsuzaki,Y.; Sugawara,K.; Furuse,Y.; Shimotai,Y.; Hongo,S.; Oshitani,H.; Mizuta,K.; Nishimura,H.
EPI603685	HE	Japan	2014-Jun-17	C/Yamagata/32/2014		Other Database Import	Tanaka,S.; Aoki,Y.; Matoba,Y.; Yahagi,K.; Mizuta,K.; Itagaki,T.; Katsushima,F.; Katsushima,Y.; Matsuzaki,Y.; Sugawara,K.; Furuse,Y.; Shimotai,Y.; Hongo,S.; Oshitani,H.; Mizuta,K.; Nishimura,H.
EPI603686	HE	Japan	2014-Jul-22	C/Yamagata/33/2014		Other Database Import	Tanaka,S.; Aoki,Y.; Matoba,Y.; Yahagi,K.; Mizuta,K.; Itagaki,T.; Katsushima,F.; Katsushima,Y.; Matsuzaki,Y.; Sugawara,K.; Furuse,Y.; Shimotai,Y.; Hongo,S.; Oshitani,H.; Mizuta,K.; Nishimura,H.

Segment ID	Segment	Country	Collection date	Isolate name	Originating Lab	Submitting Lab	Authors
EPI603687	HE	Japan	2014-Aug-20	C/Yamagata/35/2014		Other Database Import	Tanaka,S.; Aoki,Y.; Matoba,Y.; Yahagi,K.; Mizuta,K.; Itagaki,T.; Katsushima,F.; Katsushima,Y.; Matsuzaki,Y.; Sugawara,K.; Furuse,Y.; Shimotai,Y.; Hongo,S.; Oshitani,H.; Mizuta,K.; Nishimura,H.
EPI603688	HE	Japan	2006-Aug-28	C/Yamagata/14/2006		Other Database Import	Tanaka,S.; Aoki,Y.; Matoba,Y.; Yahagi,K.; Mizuta,K.; Itagaki,T.; Katsushima,F.; Katsushima,Y.; Matsuzaki,Y.; Sugawara,K.; Furuse,Y.; Shimotai,Y.; Hongo,S.; Oshitani,H.; Mizuta,K.; Nishimura,H.
EPI603689	HE	Japan	2008-Jun-25	C/Yamagata/18/2008		Other Database Import	Tanaka,S.; Aoki,Y.; Matoba,Y.; Yahagi,K.; Mizuta,K.; Itagaki,T.; Katsushima,F.; Katsushima,Y.; Matsuzaki,Y.; Sugawara,K.; Furuse,Y.; Shimotai,Y.; Hongo,S.; Oshitani,H.; Mizuta,K.; Nishimura,H.
EPI711148	HE	Australia	2012-Aug-13	C/Victoria/2/2012	Victorian Infectious Diseases Reference Laboratory	WHO Collaborating Centre for Reference and Research on Influenza	
EPI813652	HE	Japan	2002-Feb-07	C/Yamagata/5/2002		Other Database Import	Matsuzaki,Y.; Sugawara,K.; Furuse,Y.; Shimotai,Y.; Hongo,S.; Oshitani,H.; Mizuta,K.; Nishimura,H.
EPI813653	HE	Japan	2002-Jan-24	C/Miyagi/4/2002		Other Database Import	Matsuzaki,Y.; Sugawara,K.; Furuse,Y.; Shimotai,Y.; Hongo,S.; Oshitani,H.; Mizuta,K.; Nishimura,H.
EPI813654	HE	Japan	2002-Feb-22	C/Miyagi/11/2002		Other Database Import	Matsuzaki,Y.; Sugawara,K.; Furuse,Y.; Shimotai,Y.; Hongo,S.; Oshitani,H.; Mizuta,K.; Nishimura,H.
EPI813655	HE	Japan	2002-Mar-17	C/Miyagi/20/2002		Other Database Import	Matsuzaki,Y.; Sugawara,K.; Furuse,Y.; Shimotai,Y.; Hongo,S.; Oshitani,H.; Mizuta,K.; Nishimura,H.
EPI813656	HE	Japan	2002-Apr-22	C/Miyagi/28/2002		Other Database Import	Matsuzaki,Y.; Sugawara,K.; Furuse,Y.; Shimotai,Y.; Hongo,S.; Oshitani,H.; Mizuta,K.; Nishimura,H.

Segment ID	Segment	Country	Collection date	Isolate name	Originating Lab	Submitting Lab	Authors
EPI813657	HE	Japan	2002-Jun-18	C/Miyagi/31/2002		Other Database Import	Matsuzaki,Y.; Sugawara,K.; Furuse,Y.; Shimotai,Y.; Hongo,S.; Oshitani,H.; Mizuta,K.; Nishimura,H.
EPI813658	HE	Japan	2004-May-14	C/Yamagata/18/2004		Other Database Import	Matsuzaki,Y.; Sugawara,K.; Furuse,Y.; Shimotai,Y.; Hongo,S.; Oshitani,H.; Mizuta,K.; Nishimura,H.
EPI813660	HE	Japan	2004-Mar-31	C/Miyagi/25/2004		Other Database Import	Matsuzaki,Y.; Sugawara,K.; Furuse,Y.; Shimotai,Y.; Hongo,S.; Oshitani,H.; Mizuta,K.; Nishimura,H.
EPI813661	HE	Japan	2004-Jun-18	C/Miyagi/47/2004		Other Database Import	Matsuzaki,Y.; Sugawara,K.; Furuse,Y.; Shimotai,Y.; Hongo,S.; Oshitani,H.; Mizuta,K.; Nishimura,H.
EPI813662	HE	Japan	2005-Jun-07	C/Yamagata/1/2005		Other Database Import	Matsuzaki,Y.; Sugawara,K.; Furuse,Y.; Shimotai,Y.; Hongo,S.; Oshitani,H.; Mizuta,K.; Nishimura,H.
EPI813663	HE	Japan	2005-Jul-05	C/Yamagata/2/2005		Other Database Import	Matsuzaki,Y.; Sugawara,K.; Furuse,Y.; Shimotai,Y.; Hongo,S.; Oshitani,H.; Mizuta,K.; Nishimura,H.
EPI813664	HE	Japan	2005-Sep-07	C/Yamagata/3/2005		Other Database Import	Matsuzaki,Y.; Sugawara,K.; Furuse,Y.; Shimotai,Y.; Hongo,S.; Oshitani,H.; Mizuta,K.; Nishimura,H.
EPI813665	HE	Japan	2005-Apr-07	C/Miyagi/2/2005		Other Database Import	Matsuzaki,Y.; Sugawara,K.; Furuse,Y.; Shimotai,Y.; Hongo,S.; Oshitani,H.; Mizuta,K.; Nishimura,H.
EPI813666	HE	Japan	2005-May-16	C/Miyagi/4/2005		Other Database Import	Matsuzaki,Y.; Sugawara,K.; Furuse,Y.; Shimotai,Y.; Hongo,S.; Oshitani,H.; Mizuta,K.; Nishimura,H.
EPI813667	HE	Japan	2005-Dec-28	C/Fukuoka/1/2005		Other Database Import	Matsuzaki,Y.; Sugawara,K.; Furuse,Y.; Shimotai,Y.; Hongo,S.; Oshitani,H.; Mizuta,K.; Nishimura,H.
EPI813668	HE	Japan	2006-Mar-08	C/Fukuoka/2/2006		Other Database Import	Matsuzaki,Y.; Sugawara,K.; Furuse,Y.; Shimotai,Y.; Hongo,S.; Oshitani,H.; Mizuta,K.; Nishimura,H.
EPI813669	HE	Japan	2006-Jan-23	C/Yamagata/1/2006		Other Database Import	Matsuzaki,Y.; Sugawara,K.; Furuse,Y.; Shimotai,Y.; Hongo,S.; Oshitani,H.; Mizuta,K.; Nishimura,H.

Segment ID	Segment	Country	Collection date	Isolate name	Originating Lab	Submitting Lab	Authors
EPI813670	HE	Japan	2006-Jun-13	C/Yamagata/9/2006		Other Database Import	Matsuzaki,Y.; Sugawara,K.; Furuse,Y.; Shimotai,Y.; Hongo,S.; Oshitani,H.; Mizuta,K.; Nishimura,H.
EPI813671	HE	Japan	2006-Aug-08	C/Yamagata/13/2006		Other Database Import	Matsuzaki,Y.; Sugawara,K.; Furuse,Y.; Shimotai,Y.; Hongo,S.; Oshitani,H.; Mizuta,K.; Nishimura,H.
EPI813672	HE	Japan	2006-Nov-24	C/Yamagata/20/2006		Other Database Import	Matsuzaki,Y.; Sugawara,K.; Furuse,Y.; Shimotai,Y.; Hongo,S.; Oshitani,H.; Mizuta,K.; Nishimura,H.
EPI813673	HE	Japan	2006-Jun-04	C/Miyagi/4/2006		Other Database Import	Matsuzaki,Y.; Sugawara,K.; Furuse,Y.; Shimotai,Y.; Hongo,S.; Oshitani,H.; Mizuta,K.; Nishimura,H.
EPI813674	HE	Japan	2007-Jan-17	C/Yamagata/1/2007		Other Database Import	Matsuzaki,Y.; Sugawara,K.; Furuse,Y.; Shimotai,Y.; Hongo,S.; Oshitani,H.; Mizuta,K.; Nishimura,H.
EPI813675	HE	Japan	2008-Mar-26	C/Yamagata/3/2008		Other Database Import	Matsuzaki,Y.; Sugawara,K.; Furuse,Y.; Shimotai,Y.; Hongo,S.; Oshitani,H.; Mizuta,K.; Nishimura,H.
EPI813676	HE	Japan	2008-Apr-30	C/Yamagata/11/2008		Other Database Import	Matsuzaki,Y.; Sugawara,K.; Furuse,Y.; Shimotai,Y.; Hongo,S.; Oshitani,H.; Mizuta,K.; Nishimura,H.
EPI813677	HE	Japan	2008-May-19	C/Yamagata/16/2008		Other Database Import	Matsuzaki,Y.; Sugawara,K.; Furuse,Y.; Shimotai,Y.; Hongo,S.; Oshitani,H.; Mizuta,K.; Nishimura,H.
EPI813678	HE	Japan	2008-Feb-21	C/Miyagi/2/2008		Other Database Import	Matsuzaki,Y.; Sugawara,K.; Furuse,Y.; Shimotai,Y.; Hongo,S.; Oshitani,H.; Mizuta,K.; Nishimura,H.
EPI813679	HE	Japan	2010-Apr-12	C/Miyagi/3/2010		Other Database Import	Matsuzaki,Y.; Sugawara,K.; Furuse,Y.; Shimotai,Y.; Hongo,S.; Oshitani,H.; Mizuta,K.; Nishimura,H.
EPI813680	HE	Japan	2010-Apr-23	C/Tokyo/3/2010		Other Database Import	Matsuzaki,Y.; Sugawara,K.; Furuse,Y.; Shimotai,Y.; Hongo,S.; Oshitani,H.; Mizuta,K.; Nishimura,H.
EPI813681	HE	Japan	2012-Jan-16	C/Yamagata/1/2012		Other Database Import	Matsuzaki,Y.; Sugawara,K.; Furuse,Y.; Shimotai,Y.; Hongo,S.; Oshitani,H.; Mizuta,K.; Nishimura,H.

Segment ID	Segment	Country	Collection date	Isolate name	Originating Lab	Submitting Lab	Authors
EPI813682	HE	Japan	2012-Feb-07	C/Yamagata/3/2012		Other Database Import	Matsuzaki,Y.; Sugawara,K.; Furuse,Y.; Shimotai,Y.; Hongo,S.; Oshitani,H.; Mizuta,K.; Nishimura,H.
EPI813683	HE	Japan	2012-Mar-14	C/Yamagata/7/2012		Other Database Import	Matsuzaki,Y.; Sugawara,K.; Furuse,Y.; Shimotai,Y.; Hongo,S.; Oshitani,H.; Mizuta,K.; Nishimura,H.
EPI813684	HE	Japan	2012-Jun-01	C/Yamagata/14/2012		Other Database Import	Matsuzaki,Y.; Sugawara,K.; Furuse,Y.; Shimotai,Y.; Hongo,S.; Oshitani,H.; Mizuta,K.; Nishimura,H.
EPI813685	HE	Japan	2012-Jul-12	C/Yamagata/28/2012		Other Database Import	Matsuzaki,Y.; Sugawara,K.; Furuse,Y.; Shimotai,Y.; Hongo,S.; Oshitani,H.; Mizuta,K.; Nishimura,H.
EPI813686	HE	Japan	2012-Jan-26	C/Miyagi/2/2012		Other Database Import	Matsuzaki,Y.; Sugawara,K.; Furuse,Y.; Shimotai,Y.; Hongo,S.; Oshitani,H.; Mizuta,K.; Nishimura,H.
EPI813687	HE	Japan	2012-Apr-11	C/Fukuoka/1/2012		Other Database Import	Matsuzaki,Y.; Sugawara,K.; Furuse,Y.; Shimotai,Y.; Hongo,S.; Oshitani,H.; Mizuta,K.; Nishimura,H.
EPI813688	HE	Japan	2014-Apr-30	C/Miyagi/2/2014		Other Database Import	Matsuzaki,Y.; Sugawara,K.; Furuse,Y.; Shimotai,Y.; Hongo,S.; Oshitani,H.; Mizuta,K.; Nishimura,H.
EPI813689	HE	Japan	2014-May-17	C/Miyagi/5/2014		Other Database Import	Matsuzaki,Y.; Sugawara,K.; Furuse,Y.; Shimotai,Y.; Hongo,S.; Oshitani,H.; Mizuta,K.; Nishimura,H.
EPI813690	HE	Japan	2014-Jun-02	C/Miyagi/6/2014		Other Database Import	Matsuzaki,Y.; Sugawara,K.; Furuse,Y.; Shimotai,Y.; Hongo,S.; Oshitani,H.; Mizuta,K.; Nishimura,H.
EPI813691	HE	Japan	2014-Feb-01	C/Tokyo/1/2014		Other Database Import	Matsuzaki,Y.; Sugawara,K.; Furuse,Y.; Shimotai,Y.; Hongo,S.; Oshitani,H.; Mizuta,K.; Nishimura,H.
EPI813692	HE	Japan	2014-Apr-23	C/Tokyo/4/2014		Other Database Import	Matsuzaki,Y.; Sugawara,K.; Furuse,Y.; Shimotai,Y.; Hongo,S.; Oshitani,H.; Mizuta,K.; Nishimura,H.
